# A QSAR Study on the 4-Substituted Coumarins as Potent Tubulin Polymerization Inhibitors

**DOI:** 10.34172/apb.2020.032

**Published:** 2020-02-18

**Authors:** Leila Dinparast, Siavoush Dastmalchi

**Affiliations:** ^1^Biotechnology Research Center, Tabriz University of Medical Sciences, Tabriz, Iran.; ^2^Pharmaceutical Analysis Research Center, Tabriz University of Medical Sciences, Tabriz, Iran.; ^3^School of Pharmacy, Tabriz University of Medical Sciences, Tabriz, Iran.; ^4^Faculty of Pharmacy, Near East University, POBOX: 99138, Nicosia, North Cyprus, Mersin 10, Turkey.

**Keywords:** Coumarin, Cancer, Antiproliferative, QSAR, GA-MLR

## Abstract

***Purpose:*** Despite the discovery and synthesis of several anticancer drugs, cancer is still a major life threatening incident for human beings after cardiovascular diseases. Toxicity, severe side effects, and drug resistance are serious problems of available commercial anticancer drugs. Coumarins are synthetic and natural heterocycles that show promising antiproliferative activities against various tumors. The aim of this research is to computationally study the coumarin derivatives in order to develop reliable quantitative structure-activity relationship (QSAR) models for predicting their anticancer activities.

***Methods:*** A data set of thirty one coumarin analogs with significant antiproliferative activities toward HepG2 cells were selected from the literature. The molecular descriptors for these compounds were calculated using Dragon, HyperChem, and ACD/Labs programs. Genetic algorithm (GA) accompanied by multiple linear regression (MLR) for simultaneous feature selection and model development was employed for generating the QSAR models.

***Results:*** Based on the obtained results, the developed linear QSAR models with three and four descriptors showed good predictive power with r2 values of 0.670 and 0.692, respectively. Moreover, the calculated validation parameters for the models confirmed the reliability of the QSAR models.

***Conclusion:*** The findings of the current study could be useful for the design and synthesis of novel anticancer drugs based on coumarin structure.

## Introduction

Cancer is one of the severe life-threatening human health problems worldwide.^1,2^ Despite significant development in cancer chemotherapy in the past 50 years, cancer continues to be the second most frequent cause of death after cardiovascular diseases.^2,3^ There are numerous reports in the literature on the discovery of novel anticancer agents, but there is no single drug with 100% efficacy for the cancer treatment.^4^ Most of the clinically used drugs have limited effectiveness and selectivity, accompanied with serous toxicity, and unacceptable side effects.^5,6^ Moreover, the most common tumors show resistance against the significant number of commercially available anticancer drugs.^6^ Therefore, considerable demand for the discovery of efficient new anticancer drugs continues to exist in order to overcome the current chemotherapeutic problems in cancer treatment.^5^

Coumarin and its derivatives are important oxygen containing heterocycles which are found in natural products. Over the past decades, coumarins have attracted great attention because of their interesting biological and pharmacological activities such as anticoagulant,^7^ anti-inflammatory,^8,9^ antioxidant,^10^ antiviral,^11^ antimicrobial,^12,13^ antidepressants,^14^ and anti-HIV effects.^15-17^ Also, they are promising compounds due to their low toxicity, little drug resistance, less side effects, high bioavailability, and ease of chemical synthesis.^18^ Several studies have shown that coumarins are potential anticancer agents having growth suppressive effects on many types of cancers such as ovarian,^19^ breast,^20,21^ skin,^22^ prostate,^23^ liver,^24,25^ and pancreatic.^26,27^

Computer assisted drug design (CADD) has attracted considerable attention in modern drug discovery and development by reducing the time-consuming and expensive synthetic and biological experiments needed to achieve the required results.^28^ Quantitative structure-activity relationship (QSAR) studies as part of CADD techniques play a critical role in medicinal chemistry for the design of new therapeutically active compounds.^29-31^ QSAR studies are used for the prediction of the biological activity and may also be used for the interpretation of the mode of ligand-receptor interaction. The required time and cost spent for drug design and discovery are significantly decreased by using various QSAR techniques.^32^

In the current work, a QSAR analysis was conducted on a set of coumarin analogs for which biological activities have been reported in the literature.^33^ Using GA-MLR-based two-dimensional QSAR analysis, the cell toxicity of the studied coumarins was correlated to their structural features. Based on the obtained results, the developed linear models showed good predictive power, and can be used in designing new anticancer agents.

## Materials and Methods

### Methodology

#### Data set

The experimental IC_50_ (nM) values obtained for antiproliferative activities of coumarin derivatives (31 compounds) against HepG2 cell line, reported by Cao et al,^33^ were used in the present study. For QSAR analysis, all the biological data were converted into pIC_50_ (i.e., -log IC_50_).

#### Molecular descriptors

The 3D structures of the ligands were built by GuassView 5.0 software.^34^ The energy minimization of the structures were conducted initially using the empirical method (i.e., MM+)^35^ followed by semi-empirical technique AM1^36^ using the Polak-Ribiere algorithm included in HyperChem 7.5 software.^37^ The molecular descriptors for the fully optimized molecular structures were calculated using Dragon (version 3.0) program.^38^ Log *P* and log *D* were calculated by ACD/Labs 6.0 program^39^ while the molar refractivity, surface area, density, and polarizability were calculated using HyperChem 7.5 software. From the total different molecular descriptors calculated by Dragon software, descriptors with 50% constant values were omitted. Moreover, descriptors were pretreated to remove those with more than 0.95 correlations.^40^ These pretreatments on the descriptors were performed using R 3.2.3 software.^41^

#### Methods

Three algorithms were used for dividing the data set into train and test sets. These include Kenard-Stone, Euclidian Distance, and Activity/Property methodologies which are available in a java-based tool.^42,43^ For reducing the number of molecular descriptors, as well as selecting the appropriate features, multi linear regression (MLR) method optimized by incorporating the GA algorithm known as GA-MLR was used. This tool is a java-based graphical user interface and proposes an MLR model based on five validation parameters i.e. r^2^ , r^2^_Adjusted_, q^2^, $\bar{r}{\bar{m}}^{2}$ , and $\Delta \bar{r}{\bar{m}}^{2}$ with their default values set to > 0.6, > 0.6, > 0.6, > 0.5, and < 0.2, respectively.^44^ The GA-MLR approach was carried out with its default settings for finding the linear equations with three and four parameters. Although, GA-MLR was only applied on the train set, however for validating the generated models on the test set compounds, four criteria, i.e., Q^2^_(test)_, absolute percentage error (APE), mean absolute percentage error (MAPE), and standard deviation of error of prediction (SDEP) calculated according to equations 1, 2, 3, and 4, were used:

(1) $$Q_{\left(test\right)}^{2}=1-\;\frac{Press}{\sum_{i=1}^{N}{\left(y_{obs,\;i}-\;y_{m}\right)}^{2}}\;Press=\overset{N}{\underset{i=1}{\sum }}{\left(y_{pred,\;i}-\;y_{obs,\;i\;}\right)}^{2}$$

(2) $$APE=\;\frac{\left|pIC_{50\left(pred\right)}-pIC_{50\left(obs\right)}\right|}{\bar{pIC_{50\left(obs\right)}}}\;\times 100$$

(3) $$MAPE=\;\frac{\sum_{i=1}^{N}APE}{N}$$

(4) $$\text{SDEP=}\sqrt{\frac{\sum_{\text{i=1}}^{\text{N}}{{\text{(y}}_{\text{obs,i}}{\text{-y}}_{\text{pred,i}}\text{)}}^{\text{2}}}{\text{n}}}$$

Where, *y*_obs,I_*, pIC*_50(obs)_*, y*_pred,i_ and *pIC*_50(pred)_ are the experimental and predicted activities of an individual compound in the test set, respectively. N is the number of molecules and *y*_m_ is the mean of experimental biological activities of the compounds. PRESS is the predictive residual sum of the squares.

The applied fitness function (i.e., F) in this approach is as follow (for more details readers may be referred to the manual of the GA-MLR):

(5) $$F=\;\overset{i=k}{\underset{i=0}{\sum }}\frac{P_{i}-{\hat{P}}_{i}}{P_{i,Max}-{\hat{P}}_{i,Min}}$$

## Results and Discussion

The structures of coumarin analogs used in the current study are shown in Table 1. The size, lipophilicity, and electronic features of the substituents are different. For extracting chemical information from the data set compounds, computing a wide range of structural descriptors is essential for any successful QSAR analysis. In the various fields of chemometrics, it is clear that utilizing an effective variable selection method which results in reducing the complexity of the model, can improve the interpretability and the predictive ability of the developed model.^45-47^ For developing a QSAR model that explains the antiproliferative activities of the compounds shown in Table 1 on HepG2 cells, large number of structural parameters belonging to different classes of descriptors such as those listed in Table 2, are used.

**Table 1:** Chemical structures, experimental and predicted pIC_50_ values of coumarin analogs used in this study which data collected from Cao et al for model construction and absolute percentage errors for the developed models

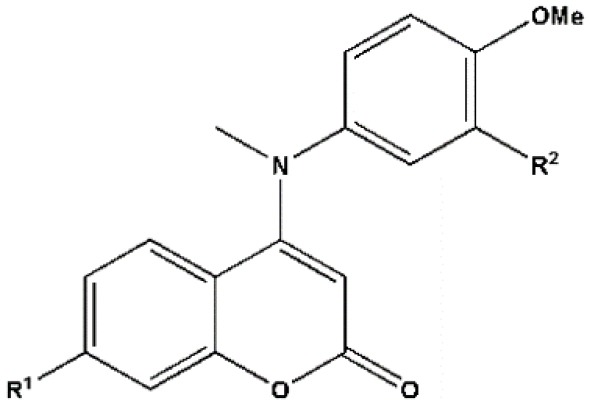						
**Compound**	**R** ^ 1 ^	**R** ^ 2 ^	**pIC**_50_ **(Exp.)**	**pIC**_50_ **(Pred.)**	**APE (Eq. 6)**	**APE (Eq. 7)**
1	OCH_3_	OH	6.406	6.340	1.021	0.431
2^*^	H	O-C(O)CH_3_	7.833	8.054	4.818	2.529
3	H	O-C(O)CHCH_2_	7.504	7.866	1.657	0.724
4	H	O-C(O)C(CH_3_)_3_	8.167	8.032	3.726	4.489
5	H	O-C(O)CH_2_Cl	8.119	7.817	3.254	3.073
6	H	O-C(O)CH_2_Br	7.963	7.703	0.051	0.556
7^*^	H	O-C(O)CH_2_CH_3_	7.889	7.615	9.305	9.618
8	H	O-C(O)(CH_2_)_3_ CH_3_	7.456	7.452	0.056	0.875
9	H	O-C(O)C(CH_2_)CH_3_	7.221	7.893	0.057	0.344
10	H	O-C(O)CHC(CH_3_)_2_	7.676	7.680	3.746	5.386
11	H	O-C(O)CHCHCH_3_	7.845	7.849	3.092	0.027
12	H	O-C(O)CCH_2_CH_2_C	7.407	7.684	0.775	1.126
13^*^	H	O-C(O)CH_2_CH_2_C(O)(O)CH_2_CH_3_	7.168	7.690	0.114	2.433
14	H	O-C(O)(CH_2_)_4_CH_3_	7.087	7.306	0.198	2.809
15	H	O-C(O)(CH_2_)_8_CH_3_	6.612	6.560	5.313	4.060
16	H	O-C(O)(CH_2_)_3_C(O)(O)CH_3_	7.565	7.557	5.367	3.592
17	H	O-C(O)(CH_2_)_3_ CH_2_Cl	7.442	7.428	0.113	2.529
18^*^	H	O-C(O)CH_2_C(CH_3_)_3_	7.140	7.209	1.088	1.399
19^*^	H	O-C(O)(CH_2_)_2_ CHCH_2_	7.629	7.509	9.202	7.212
20	H	O-C(O)C(CH_3_)CHCH_2_CH_3_	8.056	7.628	0.486	5.691
21	H	O-C(O)CHCH(CH_2_)_3_	7.991	7.562	12.410	8.225
22	H	NH_2_	8.481	8.491	2.825	2.086
23	H	HN-C(O)CH_2_CH_2_C(O)(O)CH_2_CH_3_	7.529	7.611	3.482	6.674
24	H	HN-C(O)(CH_2_)_3_C(O)(O)CH_3_	8.018	7.280	7.279	2.640
25^*^	H	HN-C(O)(CH_2_)_4_CH_3_	7.318	7.196	0.958	0.004
26	H	HN-C(O)(CH_2_)_8_CH_3_	6.543	6.574	1.569	5.705
27^*^	H	HN-C(O)(CH_2_)_3_ CH_2_Cl	7.564	7.303	1.659	1.358
28^*^	H	HN-C(O)CHCHCH_3_	7.676	7.596	3.452	0.591
29^*^	H	HN-C(O)(CH_2_)_2_ CHCH_2_	7.582	7.541	1.033	3.408
30	H	HN-C(O)C(CH_3_)CHCH_2_CH_3_	6.233	7.006	0.543	3.393
31^*^	H	OH	8.523	8.443	0.940	3.897

* Test set.

**Table 2:** Details of four most important descriptors were used in model construction

**Symbol**	**Descriptor Block**	**Description**
RDF030u	Radial Distribution Function descriptors	Radial Distribution Function - 030 / unweighted
LP1	Topological (2D matrix-based descriptors)	Lovasz-Pelikan index (leading eigenvalue)
EEig02x	Topological (Edge adjacency indices)	Eigenvalue 02 from edge adj. matrix weighted by edge degrees
Mor04p	3D-MoRSE	Signal 04 / weighted by polarizability

The total set of compounds was randomly divided into train (21 compounds, 70% of the whole data set) and test sets (10 compounds, 30% of whole data set) for the generation of QSAR models and validating the developed models, respectively. For this purpose, the hybrid methodology developed in Roy’s Lab known as GA-MLR was used on three train sets differently selected based on the three data division algorithms mentioned in Methods section. The model building processes for each set were run for thirty times for generating three-parameter models. This was led to total of 90 different models with three parameters. For all of these models the r^2^ values were compared and the best data division method was identified as being Euclidean Distance method. Then, for achieving better results on this set, twenty further runs using the same settings were performed. Moreover, for generating four-parameter equations, the best three-parameter equation was used such that the forth parameter was added one at a time from the whole pool of descriptors (i.e., in an all-walk manner) to identify the best four-parameter model. Equations 6 and 7 are the best three- and four-parameter models, respectively.

*pIC*_50_ *= 425.48937 - 0.08785 (RDF030u) -181.78082 (LP1) + 6.80549 (EEig02x)* (6)

*pIC*_50_ *= 511.93488 - 0.12088 (RDF030u) - 218.19055 (LP1) + 7.61787 (EEig02x) + 0.49323 (Mor04p)* (7)

Where *N, r*^2^ *, Q*^2^_(test)_, and MAPE are the number of compounds, the squared correlation coefficient of train set, the squared correlation coefficient of test set, and the mean absolute percentage error, respectively. Table 2 shows the statistical parameters of two developed models with more details. The numerical values and detailed information about the selected descriptors are listed in Tables 2 and 3. Correlation matrix of selected descriptors is represented in Table 4. The three-parameter model (Eq. 6) predicts the antiproliferative activities of the studied coumarins using RDF030u, LP1, and EEig02x descriptors. RDF030u (radial distribution function 3.0/unweighted) belongs to the group of Radial Distribution Function descriptors that are obtained by radial basis functions centered on different interatomic distances ranging from 0.5 to 15.5 Å.^48^ The Radial Distribution Function in a system of particles (atoms, molecules, colloids, etc), describes how density varies as a function of distance from a reference particle. When studying the chemical properties of a compound, the probability distribution of atoms scattered in a spherical volume with radius of 3.0 Å is regarded as an important factor.^49,50^ The LP1 feature, which belongs to the topological descriptors, is one of the 2D matrix-based descriptors, and is calculated by eigenvalues of a square (usually symmetric) matrix representing a molecular graph.^51^ Du and colleagues have reported that small and large values of LP1 are indicative of compounds with less and more branches, respectively.^52^ On the other hand, LP1 is a molecular branching index. The negative coefficient of LP1 in both of the developed equations indicates that the pIC_50_ is inversely related to this descriptor, which suggests that 4-substituted coumarins with lesser branches in the overall structure may be show the higher antiproliferative activity. The next feature (i.e, EEig02x), also belonging to the topological descriptors has been derived from the edge adjacency matrix weighted by edge degrees.^53^ This descriptor is associated with molecular polarity and describes the electronic effects as well as the hydrophobic properties of molecule.^54^

**Table 3:** The statistical parameters of developed models

**Models**	**N**	**r** ^ 2 ^	**r** ^ 2 ^_Adjusted_	**q** ^ 2 ^_(LOO)_	**r** ^ 2 ^ **-q** ^ 2 ^_(LOO)_	**Q** ^ 2 ^_(test)_	**SEE**	**SDEP**	$\bar{r}{\bar{m}}^{2}$	$\Delta \bar{r}{\bar{m}}^{2}$	***P*** **value**	**MAPE** **(Train set)**	**MAPE** **(Test set)**
Eq. 6	31	0.689	0.634	0.483	0.206	0.670	0.376	0.437	0.378	0.094	8.08×10^-9^	0.149	0.237
Eq. 7	31	0.749	0.686	0.530	0.210	0.692	0.349	0.416	0.417	0.138	1.89×10^-9^	0.152	0.298

**Table 4:** Correlation matrix of selected descriptors

	**EEig02x**	**LP1**	**RDF030u**	**Mor04p**
EEig02x	1			
LP1	0.24176	1		
RDF030u	0.164324	-0.06386	1	
Mor04p	0.001153	0.122393	0.571913	1

The second QSAR model (Eq. 7) describes the activities of coumarin analogs using one extra parameter added to the three previously explained features. The new parameter, i.e., Mor04p belongs to 3D-MoRSE group of descriptors, and is calculated by incorporating the polarizability-based weighting of the scattering features of the molecules.^55^ The presence of Mor04p descriptor in the developed model can be regarded as an evidence for the importance of the 3D arrangement influence of the molecules extracted from electron diffraction studies^56^ on the antiproliferative activities of the studied compounds. The increase of pIC_50_ directly correlates to the shape and size of the studied 4-substituted coumarin derivatives.

**Figure 1:**
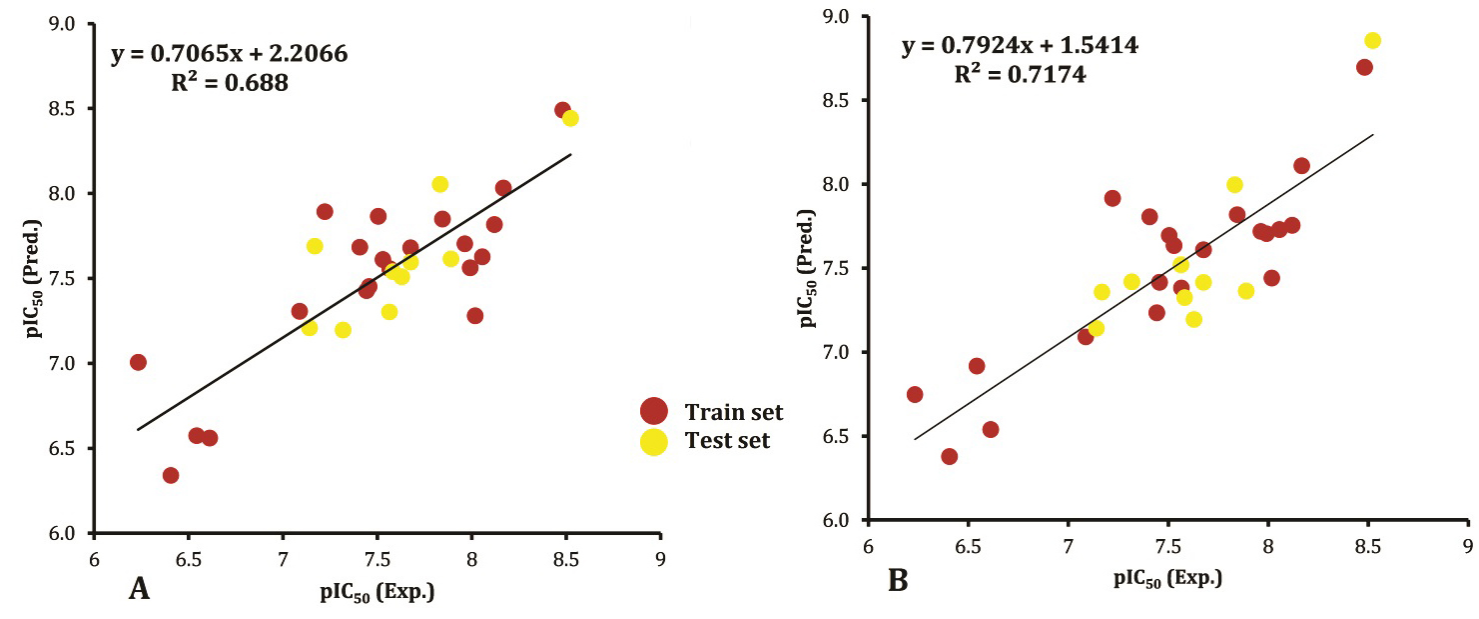
The correlation between the experimental and predicted pIC_50_ values for coumarin compounds (total data) according to Eq. 6 (**A**) and Eq. 7 (**B**).

The predictive power of the developed models was evaluated using internal and external validation measurements. For this purpose, the squared correlation coefficient (r^2^), leave one out cross-validated correlation coefficient (q^2^_(LOO)_), the squared adjusted correlation coefficient (r^2^_Adjusted_), the standard error of estimate (SEE), the SDEP, ${\bar{rm}}^{2}$, and $\Delta {\bar{rm}}^{2}$ were calculated for the train set and Q^2^_(test)_ was computed for the test set (Table 3). The squared correlation coefficient is the parameter fitted on the whole train set and the QSAR models with r^2^ > 0.6 are considered reliable.^57^ As seen in Table 3, r^2^ values of 0.689 and 0.749 were obtained for equations 6 and 7, respectively. The q^2^_(LOO)_ and r^2^ – q^2^_(LOO)_ are other measurement criteria for evaluating the performance of QSAR models, which should be higher than 0.5 and 0.3, respectively.^58-60^ The calculated values of these parameters for equations 6 and 7 are 0.483, 0.206 and 0.530, 0.210, respectively. The generated QSAR models are the result of the GA-MLR methodology based on a uni-objective (i.e., F) optimization function. The other two metrics ${\bar{rm}}^{2}\;and\;\Delta {\bar{rm}}^{2}$ were determined to further assess the predictive ability of the QSAR models. ${\bar{rm}}^{2}$ metric which was introduced by Roy and Roy determines the proximity between the observed and predicted activities for the data set.^32^ It has been suggested that for the models with reliable predictive power, the values of ${\bar{rm}}^{2}\;and\;\Delta {\bar{rm}}^{2}$ should be more than 0.5 and lower than 0.2, respectively.^61,62^ The obtained  values for equations 6 and 7 are 0.378, 0.417 and 0.094, 0.138, respectively.$\Delta {\bar{rm}}^{2}$ for both of models are in the acceptable range but ${\bar{rm}}^{2}$ values are lower than 0.5. As previously noted, the obtaining of not satisfying values is possible because in the applied GA-MLR tool was optimized based on only F function. The relatively small values of SDEPs (0.437 and 0.416) show the narrow distribution of error and indicate good performances of the proposed models for all the compounds in the train set. An important criterion for the external validation is Q^2^_(test)_ calculated for the test (unseen) set. Its value, greater than 0.5 indicates the validity of the model. In this study, Q^2^_(test)_ for the two developed models with three- and four-parameters are 0.670 and 0.691, respectively. These results demonstrate that both models have good predictive power and are reliable for the prediction of the antiproliferative activities of coumarin analogs. Furthermore, Eq. 7 has significantly higher prediction ability in comparison to Eq. 6 with *P*-value of close to zero. Figure 1 represents the correlation between the experimental and predicted pIC_50_ values according to the equations 6 and 7 for the studied coumarin compounds (total data). The resulted correlation coefficients of 0.688 and 0.717 between observed and calculated activities using Eq. 6 and Eq. 7, respectively, demonstrate the reliability of the proposed models for predictive purposes.

## Conclusion

In the present study, QSAR analysis was performed using GA-MLR method to construct models for predicting the antiproliferative activities of coumarin derivatives as potential anticancer compounds. The internal and external validation methods were used to investigate the predictive performance of the two developed MLR models. The calculated validation parameters showed that both of the models could predict biological activities of coumarins well. Based on the obtained results, the predictive power and the performance of the model with four descriptors (Eq. 7) is higher than the model with three descriptors (Eq. 6) owing to the inclusion of one more significant variable (Mor04p) in the model. Our findings could be helpful in estimating the activity as well as in designing, synthesizing, and developing the novel anticancer drugs based on coumarin scaffold.

## Ethical Issues

Not applicable.

## Conflict of Interest

Authors declare no conflict of interest in this study.

## Acknowledgments

The authors would like to acknowledge the Ministry of Health and Medical Education, and also, Biotechnology Research Center at Tabriz University of Medical Sciences for the financial support.
